# Chromosomics: Bridging the Gap between Genomes and Chromosomes

**DOI:** 10.3390/genes10080627

**Published:** 2019-08-20

**Authors:** Janine E. Deakin, Sally Potter, Rachel O’Neill, Aurora Ruiz-Herrera, Marcelo B. Cioffi, Mark D.B. Eldridge, Kichi Fukui, Jennifer A. Marshall Graves, Darren Griffin, Frank Grutzner, Lukáš Kratochvíl, Ikuo Miura, Michail Rovatsos, Kornsorn Srikulnath, Erik Wapstra, Tariq Ezaz

**Affiliations:** 1 Institute for Applied Ecology, University of Canberra, Canberra, ACT 2617, Australia; 2 Research School of Biology, Australian National University, Acton, ACT 2601, Australia; 3 Australian Museum Research Institute, Australian Museum, 1 William St Sydney, NSW 2010, Australia; 4 Institute for Systems Genomics and Department of Molecular and Cell Biology, University of Connecticut, Storrs, CT 06269, USA; 5 Departament de Biologia Cel·lular, Fisiologia i Immunologia, Universitat Autònoma de Barcelona, 08193 Cerdanyola del Vallès, Spain; 6 Genome Integrity and Instability Group, Institut de Biotecnologia i Biomedicina, Universitat Autònoma de Barcelona, 08193 Cerdanyola del Vallès, Spain; 7 Laboratório de Citogenética de Peixes, Departamento de Genética e Evolução, Universidade Federal de São Carlos, São Carlos, SP 13565-905, Brazil; 8 Graduate School of Pharmaceutical Sciences, Osaka University, Suita 565-0871, Osaka, Japan; 9 School of Life Sciences, LaTrobe University, Melbourne, VIC 3168, Australia; 10 School of Biosciences, University of Kent, Canterbury CT2 7NJ, UK; 11 School of Biological Sciences, The University of Adelaide, Adelaide, SA 5005, Australia; 12 Department of Ecology, Faculty of Science, Charles University, Viničná 7, 128 44 Prague 2, Czech Republic; 13 Amphibian Research Center, Hiroshima University, Higashi-Hiroshima 739-8526, Japan; 14 Laboratory of Animal Cytogenetics & Comparative Genomics (ACCG), Department of Genetics, Faculty of Science, Kasetsart University, Bangkok 10900, Thailand; 15 School of Natural Sciences, University of Tasmania, Hobart 7000, Australia

**Keywords:** cytogenetics, sex chromosomes, chromosome rearrangements, genome plasticity, centromere, genome biology, evolution

## Abstract

The recent advances in DNA sequencing technology are enabling a rapid increase in the number of genomes being sequenced. However, many fundamental questions in genome biology remain unanswered, because sequence data alone is unable to provide insight into how the genome is organised into chromosomes, the position and interaction of those chromosomes in the cell, and how chromosomes and their interactions with each other change in response to environmental stimuli or over time. The intimate relationship between DNA sequence and chromosome structure and function highlights the need to integrate genomic and cytogenetic data to more comprehensively understand the role genome architecture plays in genome plasticity. We propose adoption of the term ‘chromosomics’ as an approach encompassing genome sequencing, cytogenetics and cell biology, and present examples of where chromosomics has already led to novel discoveries, such as the sex-determining gene in eutherian mammals. More importantly, we look to the future and the questions that could be answered as we enter into the chromosomics revolution, such as the role of chromosome rearrangements in speciation and the role more rapidly evolving regions of the genome, like centromeres, play in genome plasticity. However, for chromosomics to reach its full potential, we need to address several challenges, particularly the training of a new generation of cytogeneticists, and the commitment to a closer union among the research areas of genomics, cytogenetics, cell biology and bioinformatics. Overcoming these challenges will lead to ground-breaking discoveries in understanding genome evolution and function.

## 1. Introduction

Advances in technology have made sequencing the entire genome of an organism essentially routine. However, DNA sequence is only one relatively static component of the highly dynamic entities within the nucleus of a cell—chromosomes. Where a particular sequence is located on a chromosome and how it interacts with other parts of the genome are important aspects of genome biology often overlooked in genome sequencing projects. We propose a new framework for studying genome biology that integrates approaches in genome sequencing, cytogenetics and cell biology, as well as a renewed focus on training the next generation of genome biologists in the skills required for the integration of these data. We propose the adoption of the term ‘chromosomics’, which combines the original definition of cytogenetics (chromosomes and cytology) with genomics (gene content, structure and function for an entire organism), to ensure a closer integration of these fields. The term chromosomics was originally proposed by Uwe Claussen to introduce the branch of cytogenetics that deals with the three-dimensional structure of chromosomes and their associated gene regulation [1]. However, we propose that this term encompass the integration of the latest advances in cytogenetics, genome sequencing, epigenomics and cell biology. The adoption of a chromosomics approach to answering the big fundamental questions in biology will undoubtedly lead to major discoveries that were previously beyond reach. 

In recent times, the field of genomics has largely distanced itself from cytogenetics, the field providing insight into chromosome structure, function and evolution. This separation of the fields has been to the detriment of a full understanding of how the genome works in the cell. These two fields were never intended to work in isolation. In 1920, Hans Winkler coined the term ‘genome’ to combine the study of genes and chromosomes [2], yet in modern interpretations of ‘genome’, chromosomes are often forgotten and the focus is solely on the DNA sequence. Similarly, Walter Sutton in 1902 (no published record) used the term ‘cytogenetics’ to combine cytology (the study of cell structure and function) with genetics (the study of genes, genetic variations and heredity). However, the cytological aspects of cytogenetics are largely ignored by most modern cytogenetic studies. As these respective fields have narrowed their focus, the result has been the development of technological and methodological advancements (examples in Table 1) that could allow us to more fully capture the dynamic nature and evolution of chromosomes from potentially any species to provide insight into fundamental biological questions.

**Figure 1:**
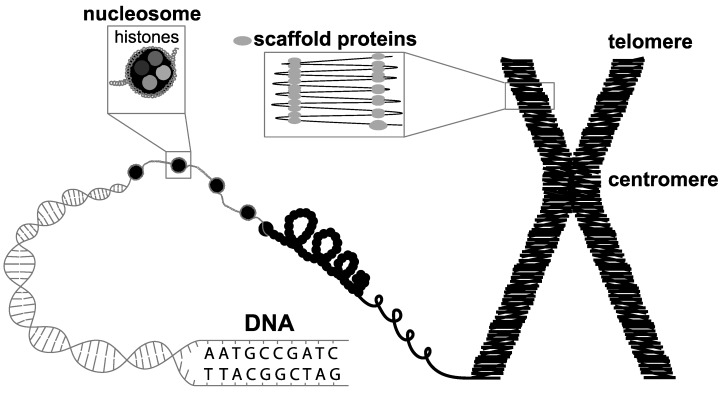
Repacking the DNA into a chromosome. The double-stranded DNA helix is wrapped around a nucleosome consisting of eight histone proteins to produce a chromatin fibre, which is attached to a backbone of non-histone proteins called scaffold proteins which form the chromosome scaffold.

Chromosomes play a vital role in the nucleus, as they are essential for DNA to replicate and segregate during cell division. They are not randomly positioned in the nucleus, but organised into specific areas called chromosomal territories [20] that change during the cell cycle [21,22] and development [23,24,25]. Maintenance of these territories is important for proper cell functioning, replication, and the accurate division and differentiation of cells. If we repack the DNA into a chromosome, we see that the DNA is wrapped around a nucleosome consisting of eight histone proteins to produce a chromatin fibre, which is attached to a backbone of non-histone proteins called the chromosome scaffold (Figure 1). The dynamic nature of the chromosome throughout the cell cycle and in response to environmental influences is enabled by the ability of the chromatin fibre to vary the level of DNA compaction and histone composition (epigenetics), and the ability of the scaffold proteins to follow the changes of the chromatin fibre [26]. Chromatin remodelling and changes in chromatin conformation affect interactions between sequences in different genomic regions and can influence gene regulation. The close connection between DNA, chromosome structure and the position of chromosomes in the nucleus highlights the need to integrate genomic data to better understand chromosome architecture and function. This information will provide a more comprehensive understanding of the evolutionary plasticity and organisational functions of genome architecture and mechanisms of faithful transmission of the genome to offspring.

**Figure 2:**
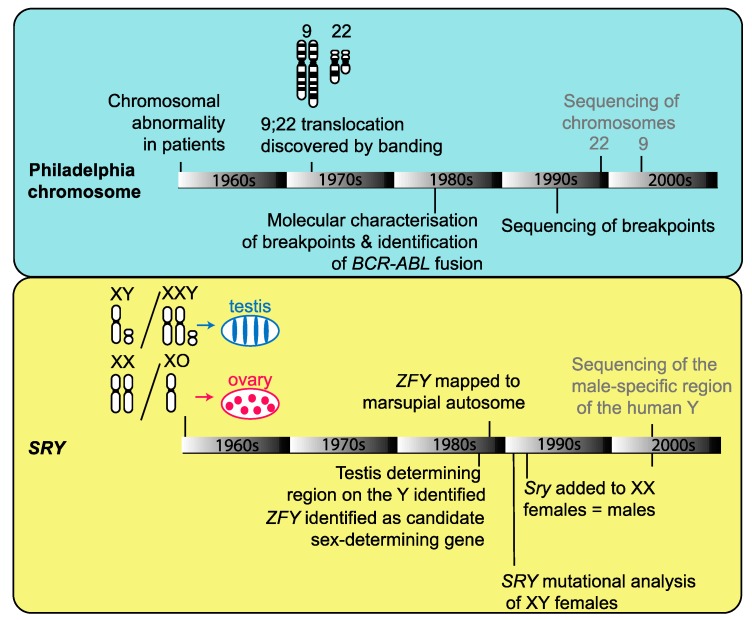
The incremental advances made through combined cytogenetic and genomic information in the discovery of the Philadelphia chromosome causing chronic myelogenous leukemia [29,30,31,32,33] and the discovery of the sex-determining gene *SRY* [34,35,36,37,38].

In the past, incremental advances in understanding genome biology have been made through combining information from cytology, cytogenetics and genomics, often from data gathered by different groups focused on one particular question and over many years (e.g., the discovery the Philadelphia chromosome causing chronic myelogenous leukemia or the discovery of the sex-determining gene, *SRY*; Figure 2). Now is the time to reunite cytogenetic and sequencing approaches. Not only has the resolution of chromosomes under various forms of microscopy greatly accelerated (e.g., deconvolution system [27], structured illumination microscopy [26] and super-resolution microscopy [28]), but new sequencing technologies are now promising to make genome assemblies close to chromosome level a reality. In addition, new sequence-based techniques for chromosome conformation capture promise to fill in the details in our cytological picture of how active and inactive chromatin is assembled and arranged into functional units in the interphase nucleus. Collectively, these advances afford the capability to answer key fundamental questions in genome biology.

## 2. Development of Genome Sequencing from Cytogenetics

The proposal to sequence the human genome led to the start of the genomics era. When we consider the human genome project, it was approached from an understanding that the position of the sequence on the chromosome was important [39]. Indeed, the forerunner of the Human Genome Project was a series of meetings of an international Human Gene Mapping consortium, which met annually to put together increasingly detailed physical maps of all the human chromosomes, combining data from linkage analysis, somatic cell genetics and radiation hybrid analysis, and in situ hybridisation. This consortium was organised into separate committees for each human chromosome, as well as committees for the mitochondrial genome. Moreover, the consortium included a comparative gene mapping committee, which started out largely focused on mouse but grew to encompass many other mammals, birds and fishes. Slowly, the physical maps developed by these working groups expanded and were filled in with other markers. Sequencing crept in to offer the ultimate detail of individual genes (or at least exomes). The advent of large insert clones like BACs (bacterial artificial chromosomes) greatly aided the extension of DNA sequence to encompass larger genomic intervals beyond individual genes [39].

Physical BAC or yeast artificial chromosome (YAC) maps of each chromosome were constructed and sequenced, resulting in a chromosome-based genome assembly and enabling the integration of gene and genetic mapping data accumulated over many years and by many different researchers [40]. The subsequent ENCODE (Encyclopedia of DNA elements) project saw the integration of sequence data with information on chromatin states, which provided an exceptional insight into dynamic gene regulation [41]. However, just as genome sequences for other species were needed to help interpret the human genome [42], comparative data from a broad range of species are required to fully understand the role many chromatin modifications play in genome function. The interpretation of the ENCODE data for the human genome was only made possible by the chromosome-based genome assembly, affording an appreciation for regional transcriptional control, dynamic chromatin states and long-range interlocus interactions. At present, a challenge for genomes from non-traditional model species is the difficulty in overlaying chromatin remodelling data when genome assemblies are not yet at the chromosome level. The platypus (*Ornithorhynchus anatinus*) genome is an excellent example of the difficulty in accurately overlaying and interpreting DNA methylation data on a fragmented genome assembly. The platypus genome was sequenced to approximately six-fold coverage by a whole genome shotgun approach using Sanger sequencing, and only around 21% of this genome assembly was anchored to platypus chromosomes [43]. Although a valuable resource, the low percentage of the genome anchored to chromosomes greatly reduced the number of genes that could be examined in a recent comparative study of reduced representation bisulphite sequencing data [44]. 

## 3. The Integral Role of Cytogenetics in Genome Projects

With increasingly cost-effective high throughput sequencing, most recently assembled genomes feature short contigs and often lack even a basic physical map or chromosome number and morphology information. While chromosome-level assemblies might not be feasible for all genomes targeted for sequencing, they should be well represented across all lineages to allow comparative genome biology studies that, by their very nature, rely on knowing the position of orthologous sequences among genomes. All genome sequencing projects should incorporate some level of cytogenetic analysis from the very start. For example, a logical first step in any whole genome sequence project would be to ensure that the individual being sequenced is not carrying chromosomal aberrations, particularly if the genome assembly is to be used as a reference for population-level sequencing. Basic karyotyping would ensure the ploidy level of the species, the absence of aneuploidy and confirm the genetic sex of the individual when cytogenetically distinguishable sex chromosomes are present, a particularly important consideration in species subject to environmental sex reversal (e.g., *Pogona vitticeps* [45]). Karyotyping will also determine if there are large heterochromatic chromosomes or regions. The flow sorting of chromosomes can be used for gross assessment of aneuploidy. Flow cytometry with appropriate standards is a reliable and fast method for estimating genome size [46], an important consideration in determining the amount of sequencing required to achieve the desired level of genome assembly.

A whole genome sequence is much more informative if it is assigned and oriented onto chromosomes, and is far more intuitive to visualise as chromosomes than unconnected and unordered scaffolds. When whole genome sequences fall short of this ‘chromosome level assembly’, their use for critical aspects of evolutionary and applied biology is significantly limited. Assigning sequence contigs to chromosomes has most often been achieved by integrating sequence data with molecular cytogenetic mapping data. This can be achieved by determining the location of a large-insert clone by fluorescence in situ hybridisation (FISH) on metaphase chromosomes or even extended chromatin fibres (fibre FISH), facilitating physical fine mapping of contigs. In Sanger sequenced genomes, this was accomplished by assigning BAC clones corresponding to individual, large sequence scaffolds. For example, the opossum genome, with a scaffold N50 (a measurement of assembly quality where 50% of scaffolds are this size or larger) of 59.8 Mb and 97% of the sequence contained in 216 scaffolds, was anchored onto the eight opossum autosomes and the X chromosome by mapping 415 BAC clones [47,48]. However, the proportion of the genome assembly assigned to chromosomes is dependent on the quality of the genome (i.e., N50 size and number of scaffolds). The platypus genome is a prime example, where the high repeat content resulted in a scaffold N50 of 957 kb, and thus only about 21% of the genome was chromosome-anchored [43]. An excellent example of where a cytogenetics approach vastly improved the accuracy of genome assembly is the tomato genome. The tomato genome, sequenced by a combination of Sanger and next generation sequencing technologies [49], benefitted greatly from the physical assignment of sequence scaffolds of BACs by FISH and confirmation by optical mapping [50]. The original tomato assembly was ordered based on a high-density linkage map. Differences in arrangement between the linkage and cytogenetic/optical maps were detected for one-third of these scaffolds, mainly in pericentric regions where a reduced level of recombination renders linkage mapping less reliable [50]. The benefits gained from assigning even a portion of the sequence to chromosomes are immense, as highlighted by the chromosomics successes listed in Table S1. 

Many genomes sequenced over the past decade have used a ‘shotgun’ approach based on short read sequence technologies to produce a series of scaffolds, often several hundred per chromosome, which are neither anchored to, nor ordered on, the chromosomes. Anchoring every scaffold to a chromosome would be a labour-intensive task, particularly if the assembly has a higher number of scaffolds; by combining computational approaches to merge scaffolds with either cytogenetic mapping and/or PCR-based scaffold verification, chromosome-level assemblies are a more achievable exercise [9,51]. In addition, the development of universal BAC clone probe sets that can be used in a high-throughput, cross-species, multiple hybridisation approach are speeding up the process of developing cytogenetic maps [9]. The advances in sequencing technology that are producing more contiguous genome assemblies, such as the contact sequencing approach of HiC-seq (e.g., Dovetail) [17], linked-read sequencing approach (10X Genomics) [12,16], long read technologies like PacBio [13] and Oxford Nanopore [15], and optical mapping (BioNano) [12], combined with high-throughput cytogenetic methods, will place chromosome-level assemblies within reach for many species. 

## 4. The Big Questions in Genome Biology Requiring a Chromosomics Approach

Despite the meticulous approach taken for the human genome, gaps remained in the genome sequence when the ‘finished’ euchromatic sequence of the human genome was published in 2004 [52]. These ‘black holes’ of the genome corresponded to the most repetitive regions such as, but not limited to, the critically important centromeres [53], nucleolar organiser regions (NORs) [54] and the Y chromosome [36]. Repetitive regions are some of the most rapidly evolving sequences and therefore, are among the most interesting regions of the genome. By employing a chromosomics approach, these hot spots of evolution are beginning to lose their black hole status in the human genome, as well as in other species. We discuss the fundamental questions arising from these evolutionary dynamic regions in relation to two overarching themes associated with genome evolution: genome plasticity and sex chromosome evolution. 

### 4.1. Genome Plasticity and Chromosome Evolution

Why do species have specific karyotypes? Why do chromosome numbers vary greatly within some groups, but are largely the same in others? Why are some of the regions of the genome so well conserved? Why are genomes so extensively changed among closely related species and others strongly conserved? Why do some chromosome rearrangements appear to lead to speciation, yet others are tolerated within a species or population of species? Is the underlying mechanism responsible for chromosomal speciation the same as that leading to chromosomal rearrangements in a disease context (i.e., cancer)? These are fundamental questions regarding genome plasticity that remain unanswered, and a chromosomics approach is essential for major breakthroughs. The answers to these questions will have wide-ranging impacts in the field of biology. Unlocking the genomic basis of speciation is a biological research priority, fuelled by the ongoing debate on species concepts and facilitated by the availability of an unprecedentedly large number of genomic resources. 

The concept of chromosomal speciation, at one stage considered a major contributor in separating populations that differ by a structural rearrangement, was virtually abandoned in favour of theories of a gradual accumulation of mutations in ‘speciation genes’ (e.g., Reference [55]). The implementation of the most recent ‘suppressed recombination model’ [56,57] has now fuelled the field using a combination of sequence and cytogenetics [58,59,60]. In this context, chromosome rearrangements could have a minimal influence on fitness, but would suppress recombination, leading to the reduction of gene flow across genomic regions and to the accumulation of incompatibilities. 

Understanding chromosomal speciation is also critical to determining the mechanism(s) underlying genome adaptation to environmental factors and how biodiversity is generated and transmitted to subsequent generations. With so many threatened species across the globe, understanding why some structural variants are tolerated within a population while others lead to reproductive isolation could prove important for the management of breeding programs for species conservation programs. In a disease context, a greater knowledge of the drivers of genome instability will aid research into human and animal diseases, particularly cancers.

Regions of genome instability can have dramatic effects for an organism. Despite being the subject of many studies using a range of species, the underlying molecular mechanisms resulting in genome restructuring/reshuffling are relatively poorly understood. For example, it remains unclear if chromosomal changes associated with speciation arise because there is an adaptive value to a specific chromosomal configuration, and what causes the genomic instability in the first place. The combined use of comparative genomics and cytogenetics of both closely and distantly related mammalian species has been extremely useful in defining models that explain genome structure and evolution [61,62,63,64,65,66,67]. Such reconstructions have revealed that the genomic regions implicated in structural evolutionary changes disrupt genomic synteny (evolutionary breakpoint regions, EBRs) and are clustered in regions more prone to breaking and reorganisation [61,62,63]. In searching for the origin (and consequences) of this evolutionary instability, approaches based purely on genome sequence have only revealed that EBRs are enriched for repetitive sequences, including segmental duplications and transposable elements, which provide the templates for non-allelic homologous recombination, resulting in inversions and additional structural changes [68,69]. Likewise, repetitive sequences in centromeric regions have been implicated in illegitimate recombination events forming Robertsonian fusions [62,70,71]. EBRs also typically occur in gene-dense regions, enriched with genes involved in adaptive processes, where changes to gene expression caused by a chromosomal rearrangement may provide a selective advantage [60,63,72]. Consequently, given the diversity of factors associated with EBRs, it is unlikely that the sequence composition of genomes is solely responsible for genomic instability during evolution and speciation.

As chromosomes are more than just DNA sequence, a more comprehensive approach that incorporates global genomic information on recombination rates, chromatin accessibility, gene function data and nuclear architecture is providing more insight into the factors underpinning genome instability. Of course, chromosome-level assemblies are an essential resource for accurate interpretation of the combination of all these data because, without such assemblies, we have a very limited (if any) understanding of the extent of the genomic restructuring that may have occurred between species, or between normal and disease states, that facilitated changes in global genomic features.

Furthermore, it has become clear that the interplay between the organisation of the genome and nuclear architecture is central to genome function [73]. We have seen a rapid evolution of methods by which to analyse genome organisation and nuclear architecture, moving from cytogenetic approaches, providing a direct measurement within individual cells of distances between loci, to chromosome conformation capture approaches (3C, 4C, 5C and Hi-C), which infer the contact among loci, typically in populations of cells rather than single cells [74,75]. However, comparison of the results obtained from chromosome conformation capture methods and FISH analyses demonstrates that care needs to be taken when interpreting data obtained solely by one method, suggesting that the use of a combined molecular and cytogenetic approach will lead to more accurate 3D models of genome organisation [76,77].

**Figure 3:**
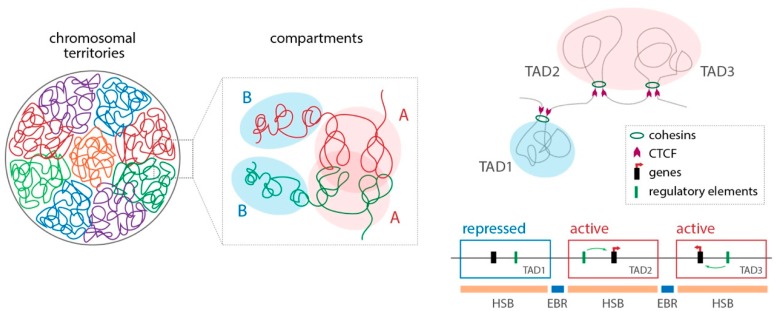
The integrative breakage model, a multilayer framework for the study of genome evolution that takes into account the high-level structural organisation of genomes and the functional constraints that accompany genome reshuffling [64]. Genomes are compartmentalised into different levels of organisation that include: (i) chromosomal territories, (ii) ‘open’ (termed ‘A’)/’closed’ (termed ‘B’) compartments inside chromosomal territories, (iii) topologically associated domains (TADs) and (iv) looping interactions. TADs, which are delimited by insulating factors such as CTCF and cohesins, harbour looping topologies that permit long-range interactions between target genes and their distal enhancers, thus providing ‘regulatory neighbourhoods’ within homologous syntenic blocks (HSBs). In this context, the integrative breakage model proposes that genomic regions involved in evolutionary reshuffling (evolutionary breakpoint regions, EBRs) which will likely be fixed within populations are (i) those that contain open chromatin DNA configurations and epigenetic features that could promote DNA accessibility and therefore genomic instability, and (ii) that do not disturb essential genes and/or gene expression.

A new model for genome rearrangements, referred to as the integrative breakage model, has recently been proposed, bringing together all of these features [64]. It posits that genome reshuffling permissiveness is influenced by (i) the physical interaction of genomic regions inside the nucleus, (ii) the accessibility of chromatin states and (iii) the maintenance of essential genes and/or their association with long-range cis-regulatory elements (Figure 3). An initial test of the integrative breakage model using rodents has supported this model [65]. EBRs were found to not only coincide with regions enriched for repetitive sequences and genes, especially genes involved in reproduction and pheromone detection, but possessed the characteristics of open, actively transcribed chromatin. The challenge remains to use a broader spectrum of species to fully test this model and dissect the underlying mechanism for chromosomal rearrangements. Such studies will now be possible with the ability to achieve chromosome-level assemblies and obtain information on chromatin modifications and nuclear architecture for non-traditional model species. A similar approach could be extended to intraspecific comparisons, such as normal versus disease samples or samples across a population where structural variants are known.

### 4.2. Sex Chromosome Evolution: Genetics and Epigenetics 

Sex chromosomes represent one of the most dynamic parts of any genome, as they are highly variable in morphology and sequence content across the plant and animal kingdoms. The special evolutionary forces experienced by sex chromosomes have rendered them highly complex entities within the genome; thus, it remains a challenge for evolutionary biologists to disentangle the varied mechanisms involved in their evolution. There are still many fundamental questions that remain unanswered because of the genomic black hole status of sex chromosomes. Why do sex chromosomes evolve and degenerate in some species but not in others? Why do sex chromosomes have a propensity to accumulate repetitive sequences that, in most cases, are species-specific? How do sex chromosomes drive speciation and hybrid incompatibilities? Why do sex chromosomes vary within a species? Why do some species have complete dosage compensation mechanisms while others do not? The complexity of sex chromosome origin, evolution and gene organisation is multilayered and cannot be understood by studying a single aspect of its biology alone. Therefore, a chromosomics approach, taking into account all aspects of cellular and molecular biology, will be essential to answering these questions. 

Many genome projects were undertaken without considering the sex chromosomes, with most projects intentionally choosing to sequence the homogametic sex in order to obtain higher sequence coverage and better assembly of the X or Z chromosome, completely neglecting to obtain sequence for the Y or W, thus ignoring the complexities of sex-delimited sex chromosome variation. Simple karyotyping with basic banding analysis, or painting one sex chromosome onto the other, can be very informative about the DNA content of the sex chromosomes. Such experiments can provide valuable information to support the adoption of appropriate sequencing technologies to obtain sequences from those unique but difficult to sequence regions of the genome. In a recent review, Tomaszkiewicz et al. [78] highlighted the need to sequence sex chromosomes, and elegantly described challenges and opportunities for combining new and emerging technologies to sequence these difficult regions of the genome. Only a chromosomics approach, combining cytogenetics and appropriate sequencing platform(s), can answer the fundamental questions regarding sex chromosome evolution. As an example, a human Y chromosome of African origin was recently assembled by flow sorting nine million Y chromosomes and sequencing using the Oxford Nanopore MinION platform, resulting in a Y chromosome assembly with an N50 of 1.46Mb [79], yet this method was much more time- and cost-efficient than that used to obtain the original human Y chromosome sequence [36,80]. 

Determining the epigenetic status of the sex chromosomes and the genes they contain is also extremely valuable. For example, the Chinese half-smooth tongue sole (*Cynoglossus semilaevis*) is a species with genetic sex determination (ZZ males and ZW females), but with a temperature override mechanism, where exposure of developing embryos to high temperatures causes genetic ZW females to develop as males (sex reversal). The sex determining gene *dmrt1* [81] is epigenetically silenced by DNA methylation in ZW females, but not in sex-reversed ZW males, where *dmrt1* expression is upregulated, leading to initiation of the male development pathway [82].

Dosage compensation, a mechanism equalizing the expression of genes on the sex chromosomes between males and females, is epigenetically controlled. A comparison of the gene content of the X chromosomes of eutherians and marsupials would suggest that a dosage compensation mechanism, in the form of X chromosome inactivation, may be shared between these two mammalian groups, as the X chromosome of marsupials is homologous to approximately two-thirds of the X of eutherians [83]. However, epigenetic analyses point to an independent evolutionary origin of X chromosome inactivation in marsupials and eutherians [84]. Similarly, there are striking differences in the extent and mechanisms of dosage compensation between more divergent taxa. For example, *Drosophila melanogaster* increases X chromosome transcription by the binding of Male Specific Lethal (MSL) complex to the single X chromosome in males to achieve dosage compensation [85]. In contrast, many species, including insects, fishes, birds, reptiles and platypus, have incomplete dosage compensation [86]. Reports of incomplete dosage compensation have most often relied purely on a sequence-based approach to measure the average transcriptional output of the X or Z chromosome between males and females for a population of cells, which does not afford an understanding of the mechanisms that impact differential transcription. However, examination of individual cells and measures of gene copies from the two Z or X chromosomes using a technique detecting nascent transcription (RNA-FISH) provides information on a single cell basis. For example, in the homogametic sex of chicken (*Gallus gallus*) and platypus, RNA-FISH detected a portion of cells expressing a gene from one copy of the X/Z, while a portion was expressed from both copies, which explains the incomplete dosage compensation pattern observed by transcriptome approaches measuring population of cells [87,88,89]. 

Sex chromosomes also have an impact beyond simply facilitating sex determination. In *Drosophila*, for example, polymorphisms in repetitive sequences on the Y chromosome influence gene expression of genes across the genome, particularly those involved in chromosome organisation and chromatin assembly [90,91]. Essentially, the polymorphic Y chromosome is a source of epigenetic variation in *Drosophila*. This epigenetic variation has implications for speciation. Engineered species hybrids showed either reduced fertility or rescued fertility depending on the origin of the Y chromosome and grandparental genetic background of the hybrid, suggesting that the Y chromosome may contribute to reproductive isolation [92]. The regulatory effect of the Y chromosome on gene expression is not limited to *Drosophila*, but has been demonstrated, at least for immune-related genes, in humans and mice [93,94]. This regulatory role for the Y highlights the importance of ascertaining not only the DNA sequence, but also the epigenetic status of sex chromosomes, in addition to, and in the context of, the rest of the genome.

## 5. Challenges Ahead for Chromosomics

Chromosomics approaches can have far greater success for answering fundamental biological questions than either genomics or cytogenetics approaches alone; this begs the question: why haven’t these two fields merged more extensively? We have identified three major challenges that may be preventing a closer union of these fields.

The biggest challenge for chromosomics is the dwindling number of researchers worldwide with expertise in cytogenetics. Rejuvenating the training of cytogeneticists is essential if the potential of chromosomics as a field is to reach its full potential. At a “Cytogenetics in the Genomics Era” workshop held in 2017 at the University of Canberra, we identified a need to renew excitement in chromosomes among undergraduate and graduate students worldwide. Genetics and genomics courses are often taught by those who have little experience in or appreciation for chromosomes, perhaps leading to anxiety in students around the study of chromosome biology. The origin of this mismatch can also be found in the backgrounds of leaders in the fields of DNA sequencing and of cytogenetics. Genome sequencing researchers often have backgrounds in biochemistry, and their training may be entirely devoid of exposure to genetics. In contrast, those who gravitate to chromosome work often have a background of zoology or botany, and may perhaps have had little exposure to biochemistry. At a time when we are more aware than ever before of the important role genome organisation and nuclear architecture plays in genome function, it is imperative that students gain an understanding of, and appreciation for, the basics of chromosome biology from their first introduction to the world of genetics and genomics. This early introduction needs to be followed by reinforcement throughout their studies. Some good courses in integrated cell and molecular biology would be a step in the right direction. 

Furthermore, graduate students have been attracted to the rapidly advancing world of genome sequencing, where mountains of data are now being rapidly and cheaply generated, as opposed to cytogenetics projects, where data are accumulated more slowly. We need to instil in students the incredible experience of observing the amazing structure of chromosomes under a microscope, and the importance of understanding chromosome structure and continuing to develop more high-throughput approaches to cytogenetics to keep pace with the rapidly advancing world of genome technology. The genomics field over the past decade has made major technological advances in obtaining genome sequences faster and more cheaply, driven mostly by the large community in this field. Advancements of a similar level in cytogenetics will require a larger community of researchers to drive the need for the technology. By increasing the training of researchers in cytogenetics, we not only increase the uptake of chromosomics, but generate a potential pool of people able to develop technologies to achieve cytogenetic analyses fast and cheaper. 

A challenge for cytogeneticists is that chromosome work, which used to be the cheapest aspect of a genome project, is now the most expensive. New sequencing technology has brought down the cost of sequencing by six orders of magnitude, and the speed at which data are generated has dramatically increased. Technical innovation in cell biology, in contrast, has greatly magnified costs. High throughput really does not apply to chromosome observation or experimentation.

The second most challenging skills area for chromosomics is the need for sophisticated bioinformatics. Whole genomes can now be rapidly sequenced, but assembling the sequence is much slower, and more labour- and computationally-intensive. Likewise, overlaying genome sequence with 3D chromatin structure data often presents a computational challenge [95]. More importantly, the incorporation of cytogenetic information with genomic data is not commonly attempted. For chromosomics approaches to be more readily applied in the future, we require more bioinformaticians to be trained generally in genome assembly, as well as with an appreciation for cytogenetics. 

Another challenge is ensuring samples, whether from wild species, laboratory species or clinical specimens, are collected appropriately for cytogenetic analysis. The collection of samples for DNA analysis is now routine, but the collection of material for the combination of cytogenetic and genomic analysis is not. The special requirements of samples collected for cytogenetic analysis need to be disseminated more widely. This is a relatively easy challenge to address by making a ‘field guide to chromosomics’ available to field researchers and a similar one to those working in a clinical setting, detailing how samples should be collected and stored for the implementation of chromosomic techniques. With the appropriate samples, a chromosomics approach could be employed to study structural and epigenetic variation at population or biogeography levels, where there is the potential to uncover the underlying genetic or epigenetic basis for adaptation to a particular environment. These data could have an impact on the conservation and management of threatened species and lead to a greater understanding of the factors underlying disease phenotypes.

## 6. Future Opportunities

### 6.1. Sequencing Genome Black Holes

Resolving highly repetitive regions of genomes, i.e., the black holes, is now possible, and we will soon have the capability to explore these previously under-represented regions of the genome to more fully understand their evolution and function. An important step towards understanding the evolution and function of repetitive regions has been the development of tools able to analyse and visualise these regions of the genome, such as RepeatExplorer [96]. Furthermore, sequencing of highly repetitive regions is now possible with long-read sequencing technology. For example, the centromeric sequence of the human Y chromosome has recently been assembled [97], demonstrating that nanopore long technology has the potential to fill in genome black holes. Likewise, combinations of different approaches where individual sex chromosomes are sequenced are proving successful in resolving at least the non-repetitive regions of sex chromosomes and identifying candidate sex-determining genes [98]. 

### 6.2. Spatial Chromosome Organisation 

The importance of the territorial organisation of chromosomes (chromosome territories) in plant and animal cells was proposed over a century ago by several cytologists (reviewed in Reference [3]). We are currently at a stage where the capacity to study the changes in spatial organisation in a population of cells, or even a single cell, is possible. The recent advances in chromatin analysis, coupled with next generation sequencing (e.g., Hi-C, Chromatin Interaction Analysis by Paired-End Tag Sequencing (ChIA-PET)) and 3D and 4D FISH, live-cell and super-resolution microscopy, provide opportunities to garner a more comprehensive understanding of chromosomal activities within the nucleus contributing to gene regulation, expression and ultimate phenotypic outcomes of an individual [22,23,24,25,99]. Such a chromosomics approach is already underway for the human genome with the launch of the 4D Nucleome project to understand how the changes in chromosome dynamics contribute to gene regulation in different cell types and biological states [99]. We will gain unprecedented insight into the role of the spatial organisation of chromosomes in genome evolution by extending this same approach to many more species. 

## 7. Concluding Remarks

We are on the verge of an exciting new revolution in biology, with a change from thinking of genomes as one-dimensional entities to defining the ways every component of the genome is packaged and changes through space and time. Chromosomics is the best path forward, providing one comprehensive analysis to answer complex questions in evolution and disease contexts. However, this can only be achieved if genomicists, cytogeneticists, cell biologists and bioinformaticians commit to forming a closer union for advancing this new era in genome biology. 

## Figures and Tables

**Table 1:** Recent Technological Advances in Cytogenetics and Genomics.

Technique	Uses	Example References
Chromosome microdissection and sequencing	Sequencing individual chromosomes/chromosome segments and haplotyping	[3,4,5]
Flow sorting of chromosomes and sequencing	Assigning sequences to chromosomes	[6,7]
Fibre-fluorescence in situ hybridisation (FISH)	Ordering of sequences large insert clones on a DNA fibre	[8]
Universal probe set and multiprobe slides	Rapid bacterial artificial chromosome (BAC) mapping across multiple species	[9]
Homologue-specific oligopaints	Visually distinguish single copy regions of homologous chromosomes	[10]
Super-resolution microscopy	Imaging of chromatin and nuclear organisation	[11]
BioNano	Genome mapping to improve assemblies and detect structural variations	[12]
Long-read sequencing (e.g., PacBio, Oxford Nanopore)	Improving genome assemblies, identifying structural variants	[13,14,15]
Linked-read sequencing (10X Chromium)	Phasing and improving scaffolding of genome assemblies	[16]
Hi-C sequencing and CHiA-PET	Chromatin interactions and improving genome assemblies (Hi-C)	[17,18,19]

## Supplementary Materials

The following are available online at https://www.mdpi.com/2073-4425/10/8/627/s1. Table S1: Examples of research questions answered with a chromosomics approach.
