# Supplementary material for: Chromosomics: Bridging the Gap between Genomes and Chromosomes
Source: Genes (Basel). 2019 Aug 20;10(8):627. doi: 10.3390/genes10080627 (PMC6723020; doi:10.3390/genes10080627)
Supplement: Supplementary file 1 [file genes-10-00627-s001.pdf]

Supplementary Material

Table 1. Examples of research questions answered with a chromosomics approach.

Research Question	Why chromosomics was needed?	Approach	Answer to research question	References
Does *XIST* exist in marsupials?	Poor sequence conservation of *XIST* across species	Mapping of flanking genes and re-examination of genome sequence	*Xist* does not exist<br>Led to discovery *Rsx*	[1–3]
What causes Fragile X syndrome (mental retardation)?	It started with the observation that many mentally retarded boys had X chromosome with gap in long arm	Identification of region stretched out in affected boys, mapping wrt DNA markers, positional cloning and sequencing of gene, identification of triplet repeats	Led to discovery of *FMR1* gene, and triplet repeat mutations	[4,5]
What causes Bloom's Syndrome?	Diagnosis of chromosome fragility and cancer susceptibility by high frequency of sister chromatic exchange	Positional cloning, cDNA's of genes in interval	Led to discovery of DNA repair pathways	[6,7]
What causes Tasmanian devil tumour disease?	Started with the demonstration that tumours from unrelated affected animals had the same abnormal karyotype.	ID of abnormal chromosomes by mapping, transcriptomics, genome sequencing and ID of candidate genes from breakpoints	Led to discovery of transmissible tumour, management implications	[8–10]
How did centromeres emerge during mammalian evolution?	Poor sequence information due to their association to repetitive DNA	Comparative mapping of flanking markers	Description of evolutionary "neocentromeres" and the "centromere repositioning" effect.<br>Led to discovery of<br>- functional satellite-free centromeres<br>- centric shifts	[11–14]

			- Robertsonian fusions (never detect with genomic sequencing – cause sterility in humans but also cause speciation)	
How to cost-effectively produce chromosome-based assemblies?	Sequencing is easy and cheap but assembling to chromosome-level requires mapping data that is time consuming and expensive	Used comparative genomics to identify predicted chromosome fragments.<br>Developed universal probe set<br>Used high-throughput ,cross species, multi-hybridisation approach.	Chromosome level assembly of pigeon and peregrine falcon genome.<br>Development of an approach that could be used for any animal genome.	[15]
Are mobile elements involved in epigenetic programs such as X inactivation?	To link the location of LINE elements to the X chromosome during X inactivation	Used RNA and DNA FISH of LINE elements on X chromosomes	Linked LINE element involvement during XCI	[16]
Where are ribosomal DNA (rDNA) loci located?	Development of "universal" probes for cytogenetic comparisons between species.	Conserved regions of rDNA genes used as probes for DNA FISH hybridise to chromosomes from divergent taxa, enabling an initial comparative analysis of karyotypes from different species.	Databases on the location of rDNA genes in animals and plants have been established, enabling karyotype comparisons across divergent taxa.	[17,18]
Where and what are the features of hotspots for meiotic recombination?	Recombination hotspots are influenced by chromatin structure therefore a combined sequence, epigenomic and cytogenetic	Initial studies used electron microscopy to study synaptonemal complexes for recombination nodules, followed	Features of recombination hotspots in eukaryotes, such as nucleosome-depleted region, chromatin	[19–21]

	approach is required to uncovered these regions.	more recently by chromatin immunoprecipitation and sequencing of purified regions associated with double strand breaks and cohesins.	accessibility and transcription factor binding.	

References:

1. Davidow, L.S.; Breen, M.; Duke, S.E.; Samollow, P.B.; McCarrey, J.R.; Lee, J.T. The search for a marsupial XIC reveals a break with vertebrate synteny. *Chromosom. Res.* **2007**, *15*, 137–146.
2. Hore, T.A.; Koina, E.; Wakefield, M.J.; Marshall Graves, J.A. The region homologous to the X-chromosome inactivation centre has been disrupted in marsupial and monotreme mammals. *Chromosom. Res.* **2007**, *15*, 147–161.
3. Shevchenko, A.I.; Zakharova, I.S.; Elisaphenko, E.A.; Kolesnikov, N.N.; Whitehead, S.; Bird, C.; Ross, M.; Weidman, J.R.; Jirtle, R.L.; Karamysheva, T. V.; et al. Genes flanking Xist in mouse and human are separated on the X chromosome in American marsupials. *Chromosom. Res.* **2007**, *15*, 127–136.
4. Lubs, H.A. A marker X chromosome. *Am. J. Hum. Genet.* **1969**, *21*, 231–44.
5. Nolin, S.L.; Brown, W.T.; Glicksman, A.; Houck, G.E.; Gargano, A.D.; Sullivan, A.; Biancalana, V.; Bröndum-Nielsen, K.; Hjalgrim, H.; Holinski-Feder, E.; et al. Expansion of the fragile X CGG repeat in females with premutation or intermediate alleles. *Am. J. Hum. Genet.* **2003**, *72*, 454–464.
6. Chaganti, R.S.K.; Schonberg, S.; German, J. A Manyfold Increase in Sister Chromatid Exchanges in Bloom's Syndrome Lymphocytes. *Proc. Natl. Acad. Sci.* **1974**, *71*, 5408–5412.
7. Ellis, N.A.; Groden, J.; Ye, T.Z.; Straughen, J.; Lennon, D.J.; Ciocci, S.; Proytcheva, M.; German, J. The Bloom's syndrome gene product is homologous to RecQ helicases. *Cell* **1995**, *83*, 655–666.
8. Deakin, J.E.; Bender, H.S.; Pearse, A.-M.; Rens, W.; O'Brien, P.C.M.; Ferguson-Smith, M.A.; Cheng, Y.; Morris, K.; Taylor, R.; Stuart, A.; et al. Genomic restructuring in the Tasmanian devil facial tumour: Chromosome painting and gene mapping provide clues to evolution of a transmissible tumour. *PLoS Genet.* **2012**, *8*, e1002483.
9. Murchison, E.P.; Tovar, C.; Hsu, A.; Bender, H.S.; Kheradpour, P.; Rebbeck, C.A.; Obendorf, D.; Conlan, C.; Bahlo, M.; Blizzard, C.A.; et al. The Tasmanian devil transcriptome reveals Schwann cell origins of a clonally transmissible cancer. *Science* **2010**, *327*, 84–87.
10. Taylor, R.L.; Zhang, Y.; Schöning, J.P.; Deakin, J.E. Identification of candidate genes for devil facial tumour disease tumourigenesis. *Sci. Rep.* **2017**, *7*, 8761.
11. Piras, F.M.; Nergadze, S.G.; Magnani, E.; Bertoni, L.; Attolini, C.; Khoriauli, L.; Raimondi, E.; Giulotto, E. Uncoupling of satellite DNA and centromeric function in the genus Equus. *PLoS Genet.* **2010**, *6*, e1000845.
12. Carbone, L.; Nergadze, S.G.; Magnani, E.; Misceo, D.; Francesca Cardone, M.; Roberto, R.; Bertoni, L.; Attolini, C.; Francesca Piras, M.; de Jong, P.; et al. Evolutionary movement

of centromeres in horse, donkey, and zebra. *Genomics* **2006**, *87*, 777–782.

13. Ventura, M.; Archidiacono, N.; Rocchi, M. Centromere emergence in evolution. *Genome Res.* **2001**, *11*, 595–599.
14. Montefalcone, G.; Tempesta, S.; Rocchi, M.; Archidiacono, N. Centromere repositioning. *Genome Res.* **1999**, *9*, 1184–1188.
15. Damas, J.; O'Connor, R.; Farré, M.; Lenis, V.P.E.; Martell, H.J.; Mandawala, A.; Fowler, K.; Joseph, S.; Swain, M.T.; Griffin, D.K.; et al. Upgrading short read animal genome assemblies to chromosome level using comparative genomics and a universal probe set. *Genome Res.* **2017**, *27*, 875–884.
16. Chow, J.C.; Ciaudo, C.; Fazzari, M.J.; Mise, N.; Servant, N.; Glass, J.L.; Attreed, M.; Avner, P.; Wutz, A.; Barillot, E.; et al. LINE-1 activity in facultative heterochromatin formation during X chromosome inactivation. *Cell* **2010**, *141*, 956–969.
17. Sochorová, J.; Garcia, S.; Gálvez, F.; Symonová, R.; Kovařík, A. Evolutionary trends in animal ribosomal DNA loci: Introduction to a new online database. *Chromosoma* **2018**, *127*, 141–150.
18. Garcia, S.; Garnatje, T.; Kovařík, A. Plant rDNA database: Ribosomal DNA loci information goes online. *Chromosoma* **2012**, *121*, 389–394.
19. Dluzewska, J.; Szymanska, M.; Ziolkowski, P.A. Where to Cross Over? Defining crossover sites in plants. *Front. Genet.* **2018**, *9*, 609.
20. Tock, A.J.; Henderson, I.R. Hotspots for Initiation of Meiotic Recombination. *Front. Genet.* **2018**, *9*, 521.
21. Vara, C.; Paytuví-Gallart, A.; Cuartero, Y.; Le Dily, F.; Garcia, F.; Salvà-Castro, J.; Gómez-H, L.; Julià, E.; Moutinho, C.; Aiese Cigliano, R.; et al. Three-Dimensional genomic structure and cohesin occupancy correlate with transcriptional activity during spermatogenesis. *Cell Rep.* **2019**, *28*, 352–367.
